# Is the Improvement of CF Patients, Hospitalized for Pulmonary Exacerbation, Correlated to a Decrease in Bacterial Load?

**DOI:** 10.1371/journal.pone.0079010

**Published:** 2013-11-29

**Authors:** Pieter Deschaght, Petra Schelstraete, Leen Van Simaey, Marleen Vanderkercken, Ann Raman, Linda Mahieu, Sabine Van daele, Frans De Baets, Mario Vaneechoutte

**Affiliations:** 1 Laboratory Bacteriology Research (LBR), Faculty of Medicine & Health Sciences, Universiteit Gent, Ghent, Belgium; 2 MucoCenter Ghent, Ghent University Hospital (UZ Gent), Ghent, Belgium; Queens University Belfast, Ireland

## Abstract

**Background:**

Cystic Fibrosis (CF) patients are vulnerable to airway colonization with *Pseudomonas aeruginosa*. In case eradication fails after antibiotic treatment, patients become chronically colonized with *P. aeruginosa*, with recurrent pulmonary exacerbation, for which patients typically are hospitalized for 2 weeks and receive intravenous antibiotic treatment. Normally, improvement of the patients' health is established.

**Aim:**

Determination of the correspondence between patient improvement and changes of the *P. aeruginosa* and total bacterial load in the sputum.

**Methods:**

Eighteen CF patients with exacerbation were included for a total of 27 hospitalization episodes. At day 1, 8 and 15, inflammation and lung function parameters were determined, together with the *P. aeruginosa* load in the sputum using culture, quantitative PCR (qPCR) and propidium monoazide qPCR.

**Results:**

Patients improved during hospitalization (decrease in levels of C-reactive protein, white blood cell counts and erythrocyte sedimentation rate, increase of FEV_1_), reaching normal values already after one week. Also the *P. aeruginosa* load and the total bacterial load decreased during the first week of antibiotic treatment (p<0.05), except for patients with a low lung function (FEV_1_≤39.4%), for whom no significant decrease of *P. aeruginosa* was established. Comparison of culture-based and propidium monoazide qPCR-based quantification of *P. aeruginosa* showed that at the end of the treatment on average 62% of the *P. aeruginosa* cells are not cultivable, indicating that many cells are alive but dormant, or dead but still structurally intact.

**Conclusion:**

Improvement of the clinical status is accompanied with a decrease of the *P. aeruginosa* load, whereby both occur mainly during the first week of antibiotic treatment. However, for patients with a low lung function, no decrease of the *P. aeruginosa* load is observed. Comparison of detection techniques shows that a large amount of noncultivable or dead bacteria are present in the samples.

## Introduction

In cystic fibrosis (CF), the CFTR protein, which is expressed in epithelial cells, is not or only partially functional due to mutations in the Cystic Fibrosis Transmembrane Conductance Regulator (*CFTR*) gene. As a consequence, these patients are vulnerable to pulmonary bacterial infections. Although a diverse set of bacterial species can be found in the CF airways [1], 80% of the patients at the age of 25–34 are colonized with *Pseudomonas aeruginosa*, which is still the most frequently isolated pathogen in CF patients [1]. In many CF centres, the patients immediately receive aggressive antibiotic treatment aiming at early eradication in case of *P. aeruginosa* isolation in respiratory samples [2]–[5]. However, complete eradication is not always achieved and patients may become colonized. Colonized patients often have recurrent infectious pulmonary exacerbations which is associated with bronchial damage, reduced lung function, a faster decline in lung function and a decreased median survival time [6]–[10]. The cause for the increased frequency of these pulmonary exacerbations remains poorly understood. It has been shown that the increased frequency is at least partially correlated with oestrogens, the use of inhaled steroids, lower FEV_1_ and/or a high frequency of previous exacerbations [11], [12]. At the Ghent University Hospital, patients with an exacerbation are hospitalized for a period of 2 weeks and receive intravenous antibiotics (aminoglycoside and beta-lactam).

Although in general, CF patients improve during this hospitalization period, the reason for the improvement is still not well explored. In this study, we investigated whether this improvement can be correlated with a change in *P. aeruginosa* density and/or a change of the total bacterial density in the sputum.

## Materials and Methods

### Patients and sampling

This study was approved by the ethical committee of Ghent University Hospital (B67020109390). Written informed consent was obtained from all the patients >18 years, or from the parents and children older than 12 years.

All 18 patients that participated in the study were chronically colonized with *P. aeruginosa* (according to the European Consensus Criteria [2]). Clinical data and the antibiotic regime for each patient are shown in Table 1. Sputum and blood samples were collected at day 1 (prior to the start of the antibiotic treatment on the same day), day 8 and day 15 of treatment for a total of 27 hospitalization periods, and inflammation parameters (C-reactive protein (CRP), white blood cells (WBC), erythrocyte sedimentation rate (ESR) and lung function (FEV_1_ and FVC) were determined.

**Table 1:** Overview of the clinical background of the patients and the antibiotic treatment received during hospitalization.

Patient	Gender	Age	CFTR Genotype	# Episodes	Antibiotic treatment^a^					
					Macrolide	TOB^b^	COL^b^	MPM^b^	TAZ^b^	CAZ^b^
1	M	26	ΔF508/ΔF508	1	Yes	Yes	?	?	Yes	?
2	V	20	ΔF508/ΔF508	3	Yes	Yes (2, 3)^c^	Yes (1, 2, 3)	Yes (1, 2, 3)	No	No
3	M	24	ΔF508/ΔF508	2	Yes	Yes (1, 2)	No	No	Yes (1)	Yes (2)
4	M	23	ΔF508/1717–1G-A	1	Yes	Yes	No	No	Yes	No
5	M	14	ΔF508/ΔF508	2	Yes	Yes (1, 2)	No	No	Yes (1, 2)	No
6	V	28	ΔF508/ΔF508	1	Yes	Yes	No	No	No	Yes
7	M	14	ΔF508/G502X	2	Yes	Yes (1, 2)	No	No	Yes (2)	Yes (1)
8	V	31	ΔF508/ΔF508	3	Yes	Yes (1, 2, 3)	Yes (1, 2, 3)	No	Yes (3)	Yes (1, 2)
9	V	28	ΔF508/ΔF508	2	Yes	Yes (2)	No	No	Yes (2)	Yes (1)
10	M	16	ΔF508/ΔF508	1	Yes	Yes (1)	No	No	No	Yes (1)
11	M	30	ΔF508/ΔF508	2	Yes	Yes (1, 2)	No	Yes (1, 2)^e^	Yes (1)^e^	No
12	V	18	ΔF508/L927P	1	Yes	Yes	No	No	No	Yes
13	V	25	ΔF508/ΔF508	1	No	Yes^d^	Yes^d^	No	Yes	No
14	M	29	ΔF508/N1303K	1	Yes	No	Yes	No	Yes	No
15	M	34	ΔF508/S1251N	1	Yes	No	No	No	Yes^d^	No
16	V	19	ΔF508/ΔF508	1	No	Yes	No	No	Yes	No
17	V	44	ΔF508/ΔF508	1	Yes	Yes	No	No	Yes	No
18	V	22	ΔF508/ΔF508	1	Yes	Yes	No	No	Yes	No

Legend:

a: Antibiotic during hospitalization (except for the macrolide treatment).

b: TOB: tobramycin, COL: colimycine, MPM: meropenem, TAZ: tazocin, CAZ: ceftazidim.

c: Numbers between brackets: episode numbers during which the antibiotic was administered.

d: TOB was replaced by COL after one week of treatment.

e: During the first episode, TAZ was replaced by MPM after one week of treatment.

### Sample processing

Sputum samples were homogenized with Sputasol (Oxoid Ltd., Basingstoke, UK) (1∶1, vol/vol, 1 h incubation at 37°C). Samples were processed immediately after arrival at the lab, within 4 hours of sample collection.

### Microbiological culture

Homogenized sputum samples were diluted serially tenfold in physiological saline. Twenty five µl of each dilution was inoculated in triplicate on cetrimide agar plates. The *P. aeruginosa* load was determined after 72 h of incubation at 37°C in ambient atmosphere.

### Propidium monoazide based inactivation of DNA from dead cells

In order to inhibit the amplification of the DNA derived from dead bacteria in the respiratory samples, the samples were treated with propidium monoazide (PMA) [13], [14]. In order to improve the PMA cross-linking efficacy, the homogenized sputum sample was split in four parts of each 200 µl and washed 3 times (centrifugation for 5 min at 5000 *g* followed by resuspension of the pellet in saline). After the final washing step, the 4 washed aliquots were transferred to a 24-well plate and 10 µl 1 mM PMA (final concentration: 50 mM) (Biotium, Hayward, CA) was added to two 190 µl sample aliquots (for PMA-qPCR) and 10 µl saline was added to the other two 190 µl sample aliquots. After 30 min incubation in the dark on a shaker, the samples were exposed to a 500 W halogen light source for 10 min at a distance of 20 cm. During exposure, the 24-well plate was kept on ice to avoid overheating of the samples.

### DNA extraction

DNA extraction from the sputum samples was performed as described previously [15], [16]. Briefly, the sputum aliquots, pretreated or not with 1 mM PMA as described above, were pre-incubated during 1 h at 55°C with 200 µl proteinase K buffer (1 mg/ml proteinase K, 0.5% SDS, 20 mM Tris-HCl, pH 8.3), after which the DNA was extracted using the bioMérieux easyMAG Nuclisens extractor (bioMérieux, Marcy-l′Étoile, France).

### Quantitative PCR

Quantitative PCR (qPCR) was performed as described previously [16]. All qPCR formats were carried out on the LightCycler480 (Roche, Basel, Switzerland), using the LightCycler480 SYBR Green kit (Roche)(for quantification of 16S rDNA) or the LightCycler480 Probes Master kit (for *P. aeruginosa*). Primers and probes used in this study are listed in Table 2. The 16S rDNA SYBR Green kit contained 5 µl SYBR Green Master Mix, 0.3 µM of each primer and 2 µl of DNA-extract. The final reaction volume was made up to 10 μl by adding HPLC water. For the *P. aeruginosa* hydrolysis probe assay, the reaction mixture contained 5 µl Probes Master kit, 0.5 µM of each primer, 0.1 µM of hydrolysis probe and 2.5 µl of DNA-extract, and the final reaction volume was made up to 10 µl by adding HPLC water. A standard tenfold dilution series was prepared by DNA-extraction from a dense suspension of log phase *P. aeruginosa* strain PA14. The *P. aeruginosa* DNA concentration was measured using the Nanodrop D1000, and the number of *P. aeruginosa* cells (chromosomes) originally present was calculated. This standard dilution series was used in the qPCR to construct a standard curve, which enabled to relate the number of cells to Cq-values, and to calculate the number of *P. aeruginosa* cells present in the samples.

**Table 2:** Primers used in this study.

Target	Primers/Probes	Sequence (5′→3′)	Annealing Temp (°C)	Reference
Universal 16S rDNA	αβNOT (forward)	AGTTTGATCCTGGCTCAG	50	[37]
	Gamma (reversed)	ACTGCTGCCTCCCGTAGGAG	50	
*P. aeruginosa - oprL*	Forward	ACC CGA ACG CAG GCT ATG	55	[15], [16]
	Reversed	CAG GTC GGA GCT GTC GTA CTC	55	
	Hydrolysis probe	6FAM-AGAAGGTGGTGATCGCACGCAGA-BBQ		

### Statistical analysis

Statistical analysis was performed using the IBM SPSS Statistics 20 software package. Paired non-parametric tests were performed using the Wilcoxon test. *P*-values below 0.05 were considered as statistically significant. The statistical correlation between different parameters was assessed by means of the Spearman's correlation test.

## Results

During 16 months, 18 patients (nine male, median age: 24.5 years) were included for a total of 27 hospitalization episodes (Table 1). For eleven patients, there was only one hospitalization episode, while five patients had two hospitalization episodes and two patients had three. In case of multiple hospitalization episodes, the median time interval between these episodes was 6 months.

### Parameters of clinical improvement

Overall, there was a statistically significant improvement of all the parameters during the full hospitalization period (Table 3). For all parameters, the major improvement occurred during the first week of treatment (statistically significant for CRP decrease (p<0.001), WBC decrease (p = 0.007), FEV_1_ increase (p = 0.001) and FVC increase (p = 0.001)) and CRP, ESR and WBC values approached normal values. During the second week, there were no significant changes in any of these parameters. Considering the total treatment period, all parameters significantly improved.

**Table 3:** Median values (ranges) for 27 episodes of hospitalization (18 patients) for the clinical parameters obtained at days 1, 8 and 15 of hospitalization.

Parameters	Median Day 1	Median Day 8	Median Day 15
**CRP (mg/L)**	22 (1–123)	4* (1–4.7)	4* (1–3.1)
**WBC (10^3^ cells/µl)**	11.61 (6.12–32.83)	9.42* (4.93–14.80)	9.41* (4.02–14.45)
**ESR (mm/h)**	27 (4–62)	23 (2–70)	20* (2–62)
**FVC (%)**	70 (29–99)	77* (39–117)	80* (37–113)
**FEV_1_ (%)**	44 (0.16–0.82)	53* (0.19–1.03)	55* (0.19–1.00)

Significant change in comparison with day 1, *p<0.05* (Wilcoxon).

### Total bacterial load and P. aeruginosa load

**Figure 1:**
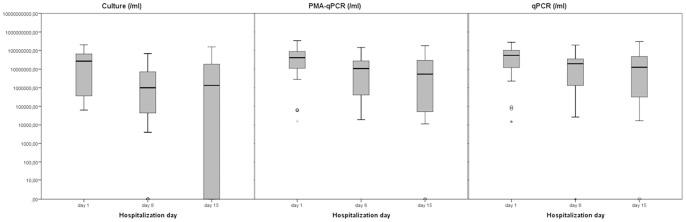
Median density (for 27 samples from 18 patients) of *P. aeruginosa* cells/ml sputum at hospitalization days 1, 8 and 15, as determined by the three quantification techniques, *i.e.* culture, qPCR and PMA-qPCR. *  =  statistically significant (p<0.05).

The change in *P. aeruginosa* sputum density during the treatment period was determined by three methods, i.e. i) quantitative culture of the bacteria on cetrimide agar, selective for *P. aeruginosa*, ii) propidium-monoazide qPCR (PMA-qPCR) and iii) qPCR, both specific for *P. aeruginosa*. Since, theoretically, PMA-qPCR only amplifies DNA of living cells (cultivable and non-cultivable), this approach is considered to provide the most relevant bacterial load estimates in this study, because culture does not detect the dormant cells and qPCR also quantifies DNA from killed cells. The PMA-qPCR median value on day 1 was 4.15×10^7^ cells/ml, whereas it was 1.08×10^7^ cells/ml on day 8 and 5.37×10^6^ cel-ls/ml on day 15 (Figure 1). For PMA-qPCR and for qPCR, the decrease of *P. aeruginosa* was only statistically significant during the first week of treatment (PMA-qPCR p = 0.002, qPCR p = 0.001) and for the total treatment period (PMA-qPCR p = 0.006, qPCR p = 0.012). According to culture results, there was a statistically significant decrease during the first week of treatment (day 1– day 8) (p = 0.03). However, during the second week of treatment (day 8– day 15) there was an increase of the amount of *P. aeruginosa* cells detected by culture (p = 0.046). Still, when considering the total treatment period (day 1– day 15), the overall decrease was significant (p = 0.037).

In this study, the values obtained with qPCR and PMA-qPCR are on average 0.5 log_10_ higher than those obtained by culture (culture vs PMA-qPCR p = 0.022, culture vs qPCR p = 0.008) (Figure 1), indicating that a significant part of the *P. aeruginosa* cells in the sputum is alive but not cultivable. However, it should be noted that part of the cells may be dead but still structurally intact, with as a result that PMA can not invade the cells and as such not inhibit the amplification of DNA of dead cells, leading to an underestimation of the number of dead cells. Moreover, PMA-qPCR values were on average 0.1 log_10_ lower than those obtained with qPCR, suggesting that part of the bacterial cells were dead/killed.

Also the total bacterial load (by means of a general 16S rRNA gene qPCR) was determined on the PMA-treated samples. Although, there was no significant decrease over the one week intervals (day 1– day 8 and day 8– day 15), the decrease over 15 days was significant (p = 0.048).

### Correlation between parameters of clinical improvement and bacterial load

During the first week of treatment, not only the parameters WBC, ESR, CRP and FEV_1_ (Table 3), but also the *P. aeruginosa* density, as determined by means of culture, PMA-qPCR and qPCR, decreased significantly (all p<0.05) (Figure 1). During the second week of treatment, no statistically significant improvements of the clinical parameters were observed as well as no statistically significant decreases of the *P. aeruginosa* load. Grouping the patients based on their FEV_1_ values at the start of the hospitalization episode (first quartile: 4 patients (for 7 episodes) with FEV_1_≤39.4%; fourth quartile: 6 patients (for 7 episodes) with FEV_1_≥73.0%) shows that for the 4^th^ quartile (FEV_1_≥73.0%), *i.e.* the patients with the best lung function, the decrease of *P. aeruginosa* load during the treatment is significant for PMA-qPCR (p = 0.011) and qPCR (p = 0.026), while no statistical significant decrease is established for the patients with a FEV_1_≤39.4% (PMA-qPCR p = 0.128, qPCR p = 0.209) (Table 4).

**Table 4:** Median and range of *P. aeruginosa* loads (cfu/ml for culture, chromosomes/ml for qPCR) of the patients, grouped based on the FEV_1_ values at the end of the treatment (Q1: FEV_1_≤39.4%, Q4: FEV_1_≥73.0%).

Quantification technique	FEV_1_ quartile	Day 1	Day 8	Day 15
**Culture**	**Q1**	1.7×10^7^	1.8×10^6^	6.2×10^5^
		6.3×10^4^–2.1×10^8^	5.8×10^4^–3.1×10^7^	0–1.4×10^8^
	**Q4**	2.6×10^7^	1.0×10^4^	0
		2.2×10^5^–8.4×10^7^	0–6.0×10^6^	0–5.0×10^7^
**PMA-qPCR**	**Q1**	7.6×10^7^	1.3×10^7^	8.9×10^6^
		1.6×10^4^–3.4×10^8^	0–5.8×10^7^	0–9.4×10^7^
	**Q4**	5.1×10^7^	6.6×10^4 a^	5.8×10^4 b^
		6.1×10^4^–1.7×10^8^	1.9×10^4^–1.7×10^7^	1.1×10^4^–3.6×10^7^
**qPCR**	**Q1**	7.2×10^7^	3.0×10^7^	2.4×10^7^
		1.5×10^4^–2.8×10^8^	0–8.3×10^7^	0–1.8×10^8^
	**Q4**	6.6×10^7^	2.2×10^5 a^	9.9×10^4 b^
		9.1×10^4^–1.7×10^8^	2.7×10^4^–4.5×10^7^	1.7×10^4^–5.8×10^7^

a: Statistical significant decrease of *P. aeruginosa* after the first week of treatment (p<0.05).

b: Statistical significant decrease of *P. aeruginosa* after two weeks of treatment (p<0.05).

### Uncultivable cells

As indicated in Figure 1, the *P. aeruginosa* values obtained by PMA-qPCR (estimating the load of all living cells) are 0.5 log_10_ higher compared to those obtained by culture (estimating the load of the cultivable cell fraction), indicating that a part of the *P. aeruginosa* bacteria is not cultivable. Considering all episodes, 41.7% of all living *P. aeruginosa* bacterial cells are not cultivable at day 1, 90.2% not at day 8 and 62.6% not at day 15 (Table 5). When comparing the patients with poor lung function (Q1, FEV_1_ value ≤39.4%) with those with a high FEV_1_ value (Q4, ≥73.0%), the former have already a high amount of not cultivable cells at the start of the treatment (5.86×10^7^/ml, 77.3%), while for the patients with good lung function only 49.1% (2.51×10^7^/ml) of the cells are not cultivable. At the end of the treatment both groups have a high proportion of dormant cells (FEV_1_ Q1: 93.1% and FEV_1_ Q4: 100%). However, the absolute values are 100-fold lower for the patients with good lung function (5.83×10^4^/ml) compared to those with poor lung function (8.32×10^6^/ml) (Table 5).

**Table 5:** Amount of dormant *P. aeruginosa* bacterial cells (/ml) in the CF sputum, i.e. number of cells obtained by means of PMA-qPCR minus the number of cells obtained by means of culturing (mean values).

	All episodes (27)	FEV_1_ Q1 (7)	FEV_1_ Q4 (7)
**Day 1**	1.93×10^7^ (41.7%)	5.86×10^7^ (77.3%)	2.51×10^7^ (49.1%)
**Day 8**	9.74×10^6^ (90.2%)	1.16×10^7^ (86.6%)	5.64×10^4^ (84.9%)
**Day 15**	3.36×10^6^ (62.6%)	8.32×10^6^ (93.1%)	5.83×10^4^ (100%)

The relative amount of dormant *P. aeruginosa* bacteria against all living *P. aeruginosa* bacteria is indicated between brackets.

## Discussion

### Intravenous antibiotic treatment improves clinical parameters

It is common practice to hospitalize CF patients with a pulmonary exacerbation for a 14-day treatment period [17]–[22]. In agreement with other studies [17]–[22], we observed an improvement of the clinical parameters of the treated patients over a 14-day treatment period. In some papers, also a 10-day period of intravenous treatment is found to be sufficient to improve the clinical parameters [23]–[27]. Indeed, our data indicate that inflammation parameters (CRP, WBC, ESR) and lung function (measured as FEV_1_ and FVC) significantly improve during the first week, with no further significant improvement during the second week. It should be noted that after one week the values had already returned to normal, such that further improvement during the second week is not expected. As stated by Plummer and Wildman [28], a shorter treatment may have several benefits, such as improvement of the life quality of the patient and a lower hospitalization cost, but they also remark that the outcome on the long term of such short treatment periods is unknown and that, for instance, the relapse time to the next exacerbation might be shorter.

### Intravenous antibiotic treatment decreases total bacterial load and P. aeruginosa load

In accordance with the change of the clinical parameters and in accordance with the culture-based study of Regelmann *et al*. [18], the major decrease of total bacterial load and of *P. aeruginosa* load occurred during the first week of treatment.

The number of bacteria determined by means of PMA-qPCR minus the number of cultivable cells allows us to estimate the number of dormant, non-cultivable cells. The load as determined by microbiological culture was on average 0.5 log_10_ lower than the load as determined by PMA-qPCR, indicating a high amount of not cultivable bacteria. This high number of dormant cells might be explained by the availability of nutrients and oxygen which decrease with the depth in the biofilm [29], [30] and which lead to a mixture of metabolically active (dividing) and metabolically inactive (dormant, non-dividing) bacteria. Is has been shown that these dormant cells are less susceptible to antibiotics and are difficult to detect by culture [30]–[32]. Taking all 27 episodes of 18 patients in consideration, we notice that after one week of treatment 90% of all *P. aeruginosa* bacteria were dormant, while at the start of the treatment only 41.7% of all *P. aeruginosa* bacteria were dormant. Although at day 15 the absolute amount of dormant cells further decreased, relatively, there were only 62.6% dormant cells. However, it should be noted that PMA can not invade dead cells which are structurally still intact, so this is still an estimation.

Taking the two outer patient quartile groups in consideration, i.e. the patients with the worst and the best FEV_1_ values, the proportion of dormant cells even increase after 15 days of treatment to 93.1% and 100%, respectively. The difference between the relative amounts at day 15 for all patients and for the two FEV_1_ outer groups might be explained by the large spread of quantities in bacterial load when all patients are included.

However, these findings illustrate that preferentially the metabolic active cells are killed during antibiotic treatment and are indicative for the need of alternative treatments targeting the dormant bacteria.

Because the load of PMA-qPCR was on average 0.1 log_10_ below that of qPCR, it can be concluded that there is a significant amount of DNA derived from dead cells present in the sputum, i.e. 23% of total *P. aeruginosa* DNA at day 0, 70% at day 8 and 42% at day 15. In agreement with the suggestion of Rogers and colleagues [33], this comparison of techniques shows that the treatment success should be determined by means of PMA-qPCR, since all viable cells are quantified (unlike culture, which does not quantify dormant cells) and since only viable cells are quantified (unlike qPCR, which also quantifies DNA from dead cells).

### Correlation between the improvement of clinical parameters and the decrease of bacterial load

In our study, we observed that clinical improvements and the decreases of *P. aeruginosa* and of total bacterial load are mainly established during the first week of antibiotic treatment.

Stressmann and colleagues [34] monitored the total number of bacteria and of *P. aeruginosa* in sputum samples of 12 adult CF patients, 21, 14, 7 and 0 days prior to a pulmonary exacerbation. Interestingly, they did not find significant changes in the bacterial load (total and/or *P. aeruginosa*) in the period prior to pulmonary exacerbation, suggesting that these exacerbations do not result from an increased bacterial density.

In some studies, the role of other bacterial species and the composition of the microbiome prior to and during pulmonary exacerbations have been identified. Using culture based techniques, Worlitzsch et al. [35] investigated the role of obligate anaerobes during pulmonary exacerbation and found that the number of obligate anaerobes was not reduced during intravenous therapy directed against *P. aeruginosa* [35]. By means of pyrosequencing, Fodor *et al.* [36] found that there is only a minimal change in the overall microbial community structure during antibiotic treatment. Moreover, they also found a similar microbiome in patients when clinical stable and during an exacerbation, suggesting that the onset of exacerbations is not correlated with a change in the airway microbiome [36].

A comparable study as ours was carried out by Regelmann *et al*. [18]. They determined the *P. aeruginosa* and total bacterial density in the sputum in an antibiotic treated group (n = 7) and in a placebo group (n = 5) and measured the lung function improvement of the patients in both groups (FVC, FEV_1_ and FEF_25–75_). In their study, reduction of the *P. aeruginosa* density was quantitatively correlated with the improvement of the above mentioned parameters (ANOVA). Our data show that both the parameters of clinical improvement and the *P. aeruginosa* load, the latter determined by means of PMA-qPCR, improve significantly only during the first week. It should be noticed that the parameters of clinical improvement reach already normal values after one week of treatment, such that further improvement during the second week is not possible. Interestingly, we found that for the 6 patients (7 episodes) with a good lung function (FEV_1_>73.0%), a significant decrease of the *P. aeruginosa* density, determined by means of PMA-qPCR could be obtained while this was not the case for the 4 patients (7 episodes) with a poor lung function (FEV_1_<39.4%). This defective clearance of *P. aeruginosa* in the latter group may be due to enhanced intrinsic resistance of the *P. aeruginosa* cells, but is probably better explained as being due to an enhanced biofilm network in the airways which makes the bacteria less reachable for the antibiotics in combination with the deterioration of the lung tissue and the immune defense of the patients.

In conclusion, we can state that the improvement of clinical status is accompanied with the decrease of the *P. aeruginosa* load, and that these decreases occur mainly during the first week of IV antibiotic treatment. Our data also indicate that the antibiotic treatment has a higher impact (regarding the decrease of the *P. aeruginosa* load) in patients with a good lung function (>73.0%). Finally, comparison of the quantitation of *P. aeruginosa* by means of culture with quantitation by means of PMA-qPCR illustrates that clearance of the dormant, non-cultivable bacteria present in the sputum is difficult to achieve with the currently applied treatments.

To determine to what extent the decrease in bacterial load could have been caused by the physiotherapy that normally accompanies antibiotic treatment, we checked bacterial loads before and after physiotherapy treatment in patients that received only physiotherapy, but could not establish a diminishment of bacterial concentrations (not sufficient patients for statistical analysis, data not published). It can be assumed that physiotherapy alone, and most certainly when combined with intravenous antibiotics, decreases the sputum in the airways, but it seems unlikely that physiotherapy alone influences the bacterial load, i.e. the number of bacteria per volume of sputum, which is in correspondence with our unpublished data.
